# Panic During COVID-19 Pandemic! A Qualitative Investigation Into the Psychosocial Experiences of a Sample of Indian People

**DOI:** 10.3389/fpsyg.2020.575491

**Published:** 2020-10-15

**Authors:** Gagan Deep Sharma, Amarpreet Singh Ghura, Mandeep Mahendru, Burak Erkut, Tavleen Kaur, Deepali Bedi

**Affiliations:** ^1^University School of Management Studies, Guru Gobind Singh Indraprastha University, New Delhi, India; ^2^FLAME University, Pune, India; ^3^State Bank Institute of Leadership, Kolkata, India; ^4^State Bank Institute of Credit and Risk Management, Gurugram, India; ^5^Department of Business, Faculty of Economics, Administrative and Social Sciences, Bahçeşehir Cyprus University, Nicosia, Cyprus; ^6^Institute for Research in Economic and Fiscal Issues (IREF), Paris, France; ^7^Department of Human Resource Management, ICFAI Business School, Gurugram, India; ^8^Sukoon Psychotherapy Center, Gurugram, India

**Keywords:** COVID-19, social psychology, semi-structured interviews, cognitive dissonance, depression

## Abstract

The outbreak of COVID-19 has spread to the entire world and is severely affecting social psychology. We conducted semi-structured interviews on 59 subjects from India to investigate the impact of information, misinfodemics (spread of wrong information), and isolation on their psychology. We perform qualitative analysis on the data. Our findings reveal that flow of information leads to anxiety, caution, and knowledge; while misinfodemics cause panic, distrust, and confusion; and isolation creates cognitive dissonance (the state of having inconsistent thoughts, beliefs, or attitudes) and adaptability among masses. The encouraging part of our findings is that, as of now, the situation is far from the state of depression. Practically, our research calls upon the government to support the masses in fighting through the crisis by focusing on pointed psychological counseling. We contribute theoretically to the body of knowledge in the field of social psychology, which is studying the psychological interventions to avoid panic amid pandemic. Future researchers in the area would do well by detailing the psychological interventions required to contain the negative impacts of the pandemic on social psychology.

## Introduction

The coronavirus disease (COVID-19), which emerged in Wuhan (China) in December 2019, has spread throughout the world, infecting 2.5 million people and causing 179,000 deaths (as on 21 April 2020) (Worldometers, 2020).^1^ COVID-19, declared a pandemic by the World Health Organization in March 2020, is generating stress among masses across the world (World Health Organization, 2020). As it is a new virus, the mechanism of action of severe acute respiratory syndrome coronavirus 2 (SARS-CoV-2) is relatively novel and no cure is currently available. COVID-19 is an ill-defined problem for the masses (Minda, 2015) and, therefore, people are likely to be influenced by fake news and myths, against which the WHO is actively coping. More than the disease itself, conventional media and social media channels are causing public stress (Depoux et al., 2020; Lima et al., 2020; World Health Organization, 2020). Given the highly contagious nature of the disease, patients are being quarantined or isolated immediately on being tested positive (Bobdey and Ray, 2020; Wang et al., 2020). Social relationships, interactions, and gatherings are integral to human life. However, due to the rapid spread of COVID-19, this critical component of human existence has been severely impacted and compromised, which has further increased stress and anxiety at the individual level. The absence of social interactions leads to overwhelming stress, depression, a state of panic, mental instability, and reluctance to work both at individual and community levels (Brooks et al., 2020). While medical and preventive interventions are of utmost importance at this stage, psychological interventions both at individual and social levels are incredibly critical as the human mind inevitably tries to bring structure to the sensory world (Minda, 2015).

Given the novelty of COVID-19 outbreak, the resultant responses, and actions needed to handle the crisis, this paper attempts to report the socio-psychological impact of the outbreak. The focus of this study is in line with Levin’s ideas of focusing on the subjective perceptions of individuals (Fiske and Taylor, 2017) rather than performing an objective analysis.

**FIGURE 1:**
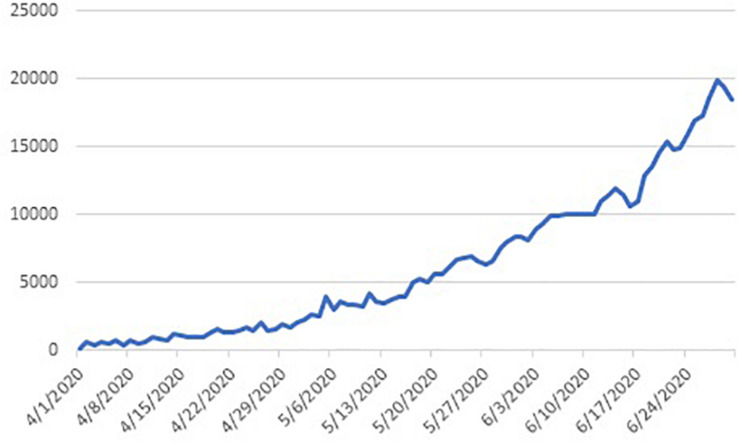
COVID-19 cases in India.

**FIGURE 2:**
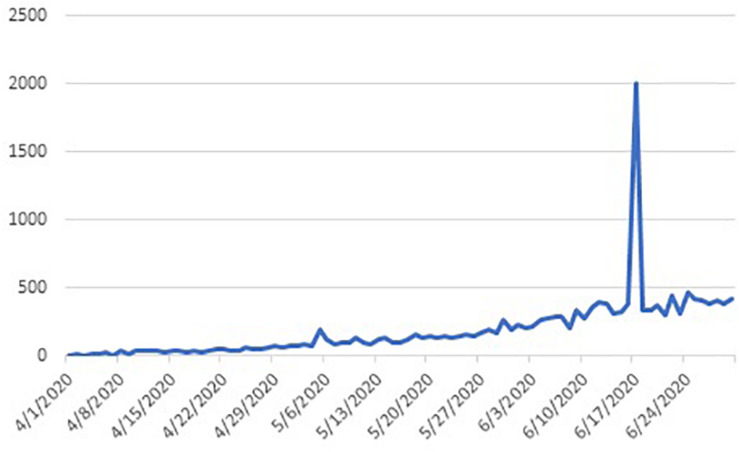
COVID-19 deaths in India.

India is the country with second largest population in the world. The outbreak of COVID-19 in India was rather late as compared to other countries, but it has picked up really fast and reached a critical stage as on date (Figures 1, 2). Given the high density of population in the country, the spread of the virus may take a threatening position for the entire world.

The study aims at capturing and measuring the psychological impact of COVID-19 on individuals and their social environment. In addition to COVID-19 infection, the world at large is experiencing mental health crisis, to which India is no exception. The major stressors associated with COVID-19 are social isolation, job loss, threat of infection, etc. These stressors are observed to have an impact on mental well-being of individuals, which leads them to approach the psychologists. Therefore, for the purpose the study, we included psychologists as the prominent category, since they would be able to provide the information not only on their own behalf but also share the experiences of their clients. In India, service sector is one of the badly effected sectors due to COVID-19, as the overseas orders have fallen significantly leading to a threat of job losses (Dhama et al., 2020). Therefore, we have given fair representation to the service sector professionals, and software experts in our study. Businessmen faced the economic consequences of this pandemic and the prolonged lockdowns by incurring unprecedented losses, due to which we included businessmen as subjects in our study. The deep correction in stock markets was observed to cause panic among the financial investors in India. To include the viewpoint of financial investors, we recruited financial consultants. We also recorded the trauma of isolation by recruiting the quarantined people. Other essential services, which were working through this period included doctors, media persons, bankers, social workers, defense personnel, insurance agents, and housekeeping staff, leading us to recruit some participants from these services. We also recruited the professionals whose work suffered heavily during this period, and their jobs were put under risk. These included educationists, artists, and graphic designers. Since spiritual healing is extensively used in India for psychological counseling, we also recruited a spiritual healer.

Based on the psychological challenges being reported by the above-mentioned classes of people, we attempt to address the following research questions.

RQ 1: What is the impact of COVID-19 information presented by media on social psychology in India?
RQ 2: What is the impact of misinfodemics (spread of an epidemic/disease through misinformation) on social psychology in India?
RQ 3: What is the impact of quarantine and isolation on the psychology of Indians battling COVID-19?

We focused on the socio-psychological impact as the understanding of people’s perception of COVID-19 is as important as the disease itself and accounts for the individuals’ construction of the situation (Fiske and Taylor, 2017). By addressing the above research questions, we make a theoretical contribution to the field of social psychology in line with the work of Levin. In addition, our findings are of practical relevance for the policymakers engaged in minimizing the negative impacts of the pandemic on social psychology in India and other nations.

Rest of the paper is organized as follows. The next section outlines the methodology of our study, the third section discusses our results, and the last section concludes.

## Materials and Methods

The methodology of our qualitative study is in line with the COnsolidated criteria for REporting Qualitative research (COREQ) guidelines. The COREQ checklist for the study is available as Supplementary Annexure 2.

### Sampling

We used a qualitative design based on semi-structured interviews (on an organic schedule) using purposive sampling to carry out our research objectives. Using the data saturation strategy as suggested by Charmaz (2014), we stopped on learning (at 59 participant) that no new information or themes are emerging in the data (Guest et al., 2006, p. 59).

One of the authors of this paper is a clinical psychologist by profession, and had information about the psychological counsellors who were providing counseling services to the above categories of people during the pandemic. Through this network, key counsellors were identified and recruited for the study. Using the leads from these counsellors, as also some other relevant networks, professionals from other sectors were contacted through emails (as the physical interviewing was not possible due to lockdown). Our respondents included the following–

**Table:** 

Psychologists	12
Service sector professionals	09
Software experts	09
Businesspersons	04
Financial consultants	03
COVID-19 positive and quarantined	02
COVID-19 negative but quarantined	04
Media professionals	03
Artists	02
Educationists	02
Social workers	02
Banker	01
Insurance advisor	01
Spiritual healer	01
Doctor	01
Graphic designer	01
Housekeeping staff	01
Defense personnel	01

### Data Collection

The data were collected by a professional clinical psychologist (details available in Supplementary Annexure 2). As deductive qualitative approach allows the inclusion of many different kinds of data collection and analysis techniques, we used a thematic analysis to gain an in-depth understanding of the phenomenon of interest. The qualitative approach adopted was to delineate the psychological impact of isolation and COVID-19 information provided by media on social psychology in India.

Semi-structured interviews were conducted with 59 selected participants from India. The respondents were aware about SARS-CoV-2 and the COVID-19 pandemic and are under the state of lockdown since 26 March 2020. We interviewed the participants and asked open-ended questions expanded from the primary research questions. The participants expressed their experiences, views, and feelings about the impact of isolation as well as COVID-19 information presented by media. The interviews lasted for 30–45 min and were audio-recorded and transcribed verbatim.

### Data Coding/Code Development

The unit of analysis was “interviews.” The coding unit “sentences” was used throughout the coding phase. An attempt was made to establish relevance based on meaning rather than just frequency. The coding units were copied and pasted to the memo “coding process” and were read and checked for sense and contextuality. Following the five elements of a good thematic code, a code book was written to define each code using a label (name), definition of the theme, description that flags when the theme is likely to occur, inclusion and exclusion criteria, and examples of occurrences of the theme (refer to Supplementary Annexure 1) (Boyatzis, 1998). The coding scheme used was theory-driven.

### Data Analysis

Deductive pattern seeking was used as a method of scientific reasoning. Within this context, we proposed that COVID-19 information presented by media leads to anxiety and knowledge enrichment among viewers, while isolation leads to cognitive dissonance and adaptability.

## Results and Discussion

Our study focused on examining the (a) impact of COVID-19 information presented by media on social psychology in India, (b) impact of misinfodemics (spread of an epidemic/disease through misinformation) on social psychology in India; and (c) impact of quarantine and isolation on the psychology of Indians battling COVID-19. We followed the stopping rule for qualitative investigations as advocated by Charmaz (2014). In line with Guest et al. (2006, p. 59), we continued to recruit the participants till new information or themes kept on emerging. From 57 participant, we started observing that the data saturation has arrived on all the themes other than cognitive dissonance. For the theme of cognitive dissonance (in response to the RQ3), the data saturation was observed at 59 participant, leading us to stop recruiting the participants.

**FIGURE 3:**
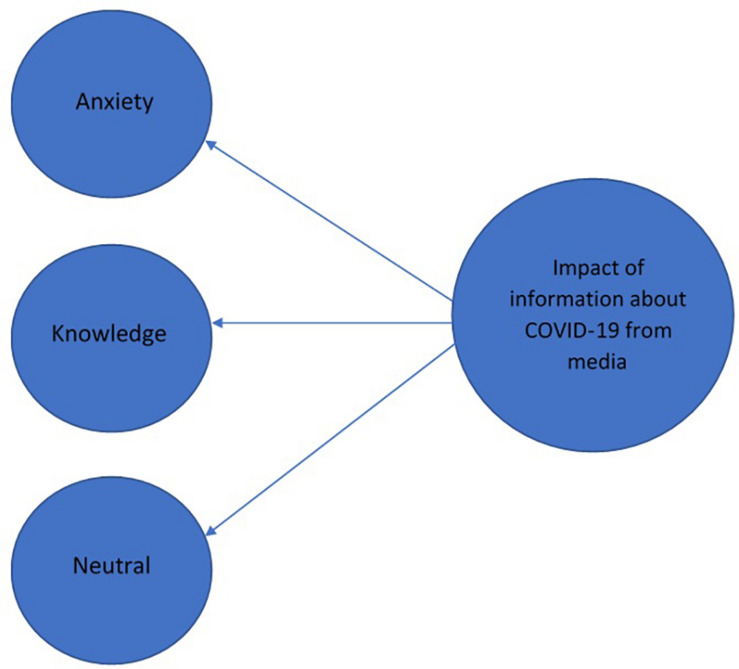
Impact of information about COVID-19 presented by media.

**FIGURE 4:**
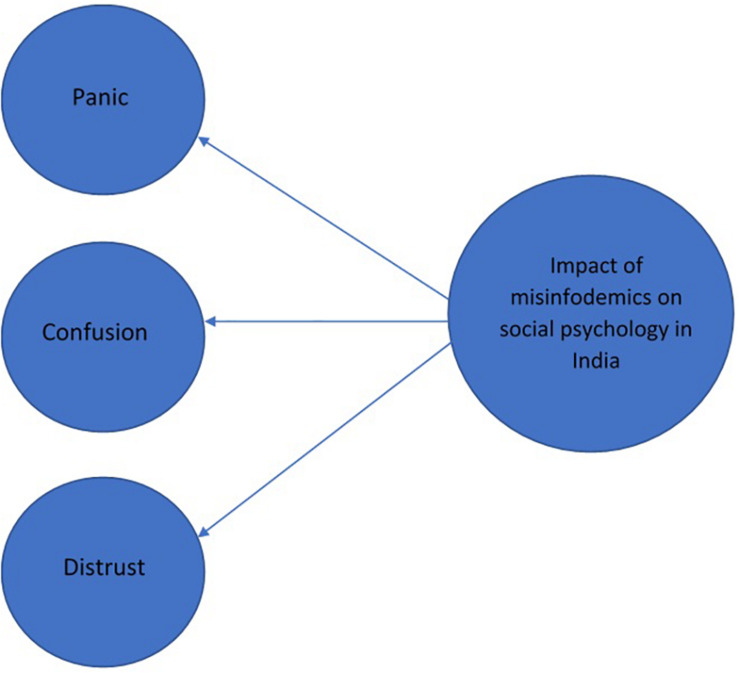
Impact of misinfodemics on social psychology in India.

**FIGURE 5:**
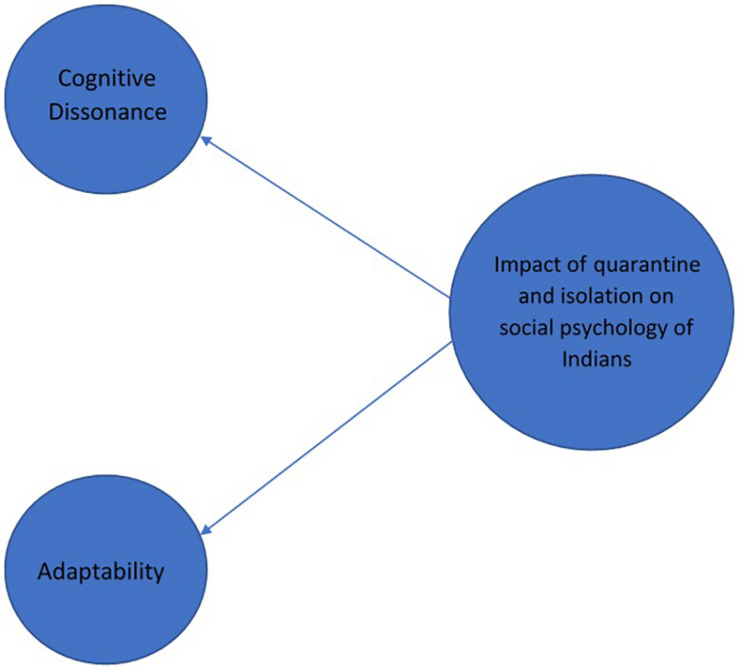
Impact of quarantine and isolation on social psychology of Indians.

In this section, we present the results and discuss them in accordance with the research questions. In addition, we present the relevant quotes from the subjects with regard to the research questions and the themes in Supplementary Annexure 1. Figures 3–5 are concept maps of the responses of subjects.

### Impact of the Information About COVID-19 Presented by Media on Social Psychology in India

Based on the responses that were received from the subjects, the impact of COVID-19 information presented by media on the social psychology in India can be classified into three themes— (a) anxiety; (b) knowledge; and (c) neutral. These themes were identified based on the responses of the subjects in Supplementary Annexure 1.

India is under lockdown (a 21-day lockdown, followed by an extension of 19 days) since 24 March 2020 (BBC News, 2020a,b), due to which the availability of physical sources of information are limited, underlining the role of media in sharing information regarding COVID-19 (Happer and Philo, 2013). Information shapes social psychology and public opinion, therefore, individuals having negative belief apropos media feel that misinformation causes anxiety and depression (Yuan et al., 2020). However, it is challenging to measure the myriad of misinformation (Nawrat, 2020). The disagreement among different sources of information leads to ambiguity in general public.

It appears that some of our respondents believe the information (Supplementary Annexure 1) and treat it as a significant contributor to their knowledge. According to them, information is a vehicle that helps masses sail through the crisis by serving as a liaison between people and the government. The information also cautions masses about the consequences of committing mistakes. Through experiential sharing, the media makes people appreciative of social distancing and makes them aware of the administrative and infrastructural arrangements. However, during these testing times, media needs to play a responsible role in creating public opinion, failing which, people may develop negative opinion toward this important pillar of society (World Health Organization, 2016).

India is broadly an informal economy filled with migrant labor (Chandrasekhar and Ghosh, 2020). The lockdown has resulted in job losses for thousands of migrant laborers, who were left with no option but to walk till their hometowns, leading to lockdown violations (Rising, 2020). Worries of the masses are further aggravated by the fear of uncertainty regarding economic security resulting from the potential job losses due to lockdown and the long-term effects of COVID-19 (Chandrasekhar and Ghosh, 2020; Goyal, 2020).

Altogether, the impact of media on the social psychology of a majority of people was found to be negative (causing anxiety), while it was neutral (causing caution) and positive (causing knowledge enrichment) in a few cases. The role of media is immensely significant during these testing times as people need to be precisely informed about the do’s and don’ts in order to be sufficiently prepared to deal with the pandemic. As a result, the negative and neutral feelings of anxiety and caution, respectively, may get transformed positively into knowledge enrichment (Fiske and Taylor, 2017). The policy interventions at the level of governmental and non-governmental bodies may be directed at ensuring the timeliness and precision of the information flow regarding COVID-19 (Roy, 2020).

### Impact of Misinfodemics on Social Psychology in India

Misinfodemics refers to the spread of false information during a pandemic with or without any maleficent intention (Drexler, 2019). While the information is reliable for the most part, it can be inaccurate at times. The spread of misinformation worsens the impact of the pathogen and creates a feeling of uncertainty amongst individuals (Drexler, 2019; Banerjee, 2020). Furthermore, the uncertainty generates ambiguity and creates a situation of (a) panic, (b) confusion, or (c) distrust among masses.

In our study, we also observed that the respondents were agitated and frustrated, which is detrimental to their mental well-being. Concurrently, amidst the pandemic, conventional public health responses are not enough to supersede these contemporary digital sources. Online connectivity makes people xenophobic toward the infected ones, and they may take wrong medications that affect their physical as well as mental well-being. It is often found that the availability of precise and timely information plays a positive role in building a harmonious situation.

The lockdown of major economic activities in India has caused a steep fall in the agricultural, manufacturing, and service activities in the country (Bloomberg, 2020a; Business Today, 2020; BusinessLine, 2020; Goyal, 2020). The spread of unreliable information about lockdown and its impact on the national economy, the resultant job losses, and slowing down of the economy further affect social psychology in the country (Madhav et al., 2017; Bloomberg, 2020b; McKibbin and Fernando, 2020; Roy, 2020). This uncertainty adds fuel to the fire by causing chaos and confusion among masses, leading to irrational decision making. For instance, the misinformation regarding working of public transport in Mumbai led to a stampede at a train station (NDTV, 2020). All these factors are responsible for causing a feeling of distrust among masses, thus significantly hampering social psychology. Similarly, in the past also, misinfodemics have affected the treatment and renormalization of depression, even leading to suicides. Certain cases of suicide have also been reported in India as a result of the panic caused by COVID-19 (Ojha, 2020). It is indispensable for media to compile and publicize accurate information, and therefore, the masses need to exert some control over the information and forward it responsibly.

### Impact of Quarantine and Isolation on the Social Psychology of Indians Battling COVID-19

Our findings reveal that quarantine and isolation are causing (a) cognitive dissonance or (b) adaptability in Indians. Amidst the pandemic, individuals are experiencing swelling of health, economic, and humanitarian crisis through every dimension of their social fabric. The way people bounce back from the state of cognitive dissonance to the state of adaptability as a result of the pandemic and restrictions resulting from the pandemic suggests that the society is moving toward a new normal Initially, the individuals were found to resist such a situation due to mobility constraints and the fear of no escape and losing their livelihood. However, in the course of time, it appears that they are willing to perform multiple tasks. Another fear among masses was the compulsion to stay together with their families without having any outlet to move out for long. This belief arouses the feeling of restlessness, confusion, frustration, and stress, due to which, people tend to lose trust in the system, leading to deterioration of their mental well-being.

On the positive side, these conditions have developed the idea of appreciative inquiry among masses, as they are able to appreciate that their captivation will effectively help in controlling the disease. Individuals, during self-quarantine, feel that social distancing has given them an opportunity for psychological explorations and developing their intelligence- and emotional-quotient. For instance, they have ample time to spend with themselves, their family, and in natural surroundings, and they are able to acknowledge the fact that psychological communication is the key to bonding. The masses are also able to admire the idea of achieving a work–life balance and look forward to innovative ways of working from home.

## Conclusion

This paper contributes to the theoretical field of social psychology by addressing the subjective perceptions of our respondents, and holds practical significance by informing the policymakers on tackling the panic amid pandemic. The semi-structured interview-based qualitative analysis conducted through this study on a sample of 59 subjects revealed that information, misinfodemics and isolation emerge as three prominent factors impacting the social psychology of Indians during the COVID-19 outbreak. We found that flow of information leads to anxiety, knowledge, and neutral approach in India. The governments may address the flow of information in interest of transparency, so that the outcomes in the form of anxiety and neutral approach may shift toward knowledge, thereby leading to management of the pandemic in a more effective manner. The sources of information in these critical times are limited, and the reliability of those is also questionable, which is reflected from the panic emerging from the misinfodemics. We suggest that to avoid panic in such critical times, the policymakers need to focus on misinfodemics, which are a result of fake news, in general. The encouraging fact of our study is that isolation is not observed to drive toward critical psychological patterns, such as depression. Rather, isolation drives Indians toward cognitive dissonance and adaptability, which is a sign of psychological strength. The governments need to plan the psychological interventions in such a way that the citizens can productively utilize the period of isolation.

## Data Availability Statement

The raw data supporting the conclusions of this article will be made available by the authors, without undue reservation, to any qualified researcher.

## Ethics Statement

Ethical review and approval was not required for the study on human participants in accordance with the local legislation and institutional requirements. The patients/participants provided their written informed consent to participate in this study.

## Author Contributions

All authors listed have made a substantial, direct and intellectual contribution to the work, and approved it for publication.

## Conflict of Interest

MM was employed by companies State Bank Institute of Leadership, Kolkata, India, and the State Bank Institute of Credit and Risk Management, Gurugram, India and DB was employed by company Sukoon Psychotherapy Center. The remaining authors declare that the research was conducted in the absence of any commercial or financial relationships that could be construed as a potential conflict of interest. The reviewer AA declared a shared affiliation with one of the authors, AG, to the handling editor at the time of review.

## Footnotes

1 www.worldometers.info
