# Supplementary material for: Panic During COVID-19 Pandemic! A Qualitative Investigation Into the Psychosocial Experiences of a Sample of Indian People
Source: Front Psychol. 2020 Oct 15;11:575491. doi: 10.3389/fpsyg.2020.575491 (PMC7594525; doi:10.3389/fpsyg.2020.575491)
Supplement: Supplementary file 1 [file Table_1.DOCX]

**Panic amid pandemic! An empirical investigation into social psychology in India during Covid-19 outbreak**

**Annexure 1**

**Selected quotations of subjects about Research Questions**

**RQ 1: What is the impact of the information about Covid-19 from media on social-psychology in India?**

Our analysis identified three themes; (1) Anxiety, (2) Knowledge and (3) Neutral

**Anxiety:**

The participants reported that Anxiety which includes feelings of fear, worry, and unease been the outcome of the media information on COVID 19:

“…It may have major impact on my finances …” Participant 3

“…Concern for my family safety and my business after corona impact cool down …” Participant 6

“…It impacted initially....but over a period of time most of us are either fed up or have gotten immune to it…” Participant 8

“… I’m disappointed and afraid of this …” Participant 11

“…Intermittently makes me feel despair…” Participant 14

“…Appreciate the lockdown and other measures by the government. Uncertainty about the future, especially economic crisis …” Participant 15

“…If there are so many hotspot areas and we are stretching the lockdown days, then it should be a big number what we are getting the numbers it could not be actual …” Participant 17

“…The uncertainty of the present time worries me about my job, education, the welfare of the family, and economic strains that it may cause in the near future. Both individually and globally and its impacts on my life …” Participant 19

“…Scares me most...and sometimes useful …” Participant 20

“…It's affecting my routine life…” Participant 26

“…I feel very negative…” Participant 28

“…There is some sort of building up of pressure…” Participant 30

“…The number of Covid-19 patients every day and their increase impact me a lot …” Participant 32

“…It triggers Panic…” Participant 35

“…I am scared …” Participant 39

“…It impacts mentally…” Participant 40

“…Information by media increases my Anxiety…” Participant 42

“…negative in few things, other than that nothing special…” Participant 44

“…It impacts mentally…” Participant 45

“…honestly, I used to read the news a lot. I have reduced doing so because the news is always so sad and concerning, scary even, yes, I do understand the importance of it …” Participant 50

“…feeling panicked sometimes, sometimes getting fidgety and irritated, wanting to blow off the people around fear for other not so known or close people who are also that in the circuit of the spread of the disease anyone might get hit by anything …” Participant 56

“…it keeps them informed it is also intellectually stimulating and exciting to discuss some of them experience anxiety about getting infected …” Participant 57

“…this is the first step to anxiety; communication is the key …” Participant 58

“…they got apprehensive and fearful key …” Participant 59

**Knowledge:**

The participants reported that increase in the Knowledge as an outcome of the media information on COVID 19:

“…Gives me Knowledge of what is happening in the world...” Participant 2

“…Just got awareness ...” Participant 4

“…Keeps me update about my responsibilities and duties as a citizen. It Makes me aware of the evolving situation and provides insights into what to expect in the near future ...” Participant 5

“…Positively, making amends to reduce risks ...” Participant 7

“…Not to go outside ...” Participant 9

“…Making aware of our surroundings...” Participant 10

“…I am prepared to fight for it ...” Participant 12

“…To an extend creates awareness of safety measures and keeps updated as to present situation as to whether it is worsening ...” Participant 13

“…Making helps to understand how to take appropriate precautionary measures to avoid further spreading of the disease ...” Participant 18

“…It keeps me updated about the trending cases and serious preventions to be taken it also updates me daily...” Participant 21

“…To plan long term and get a fair idea, where it will lead us in the near future ...” Participant 22

“…Makes me be careful and learn ways to avoid mistakes ...” Participant 24

“…Act accordingly ...” Participant 25

“…I need to understand the short-term and long-term impact of this outbreak, have access to support systems, be informed so that I can take appropriate decisions ...” Participant 27

“…Information makes us alert ...” Participant 31

“…Somewhat make you aware ...” Participant 33

“…Made me aware of the situation ...” Participant 34

“…It provides clarity ...” Participant 36

“…Aware me about the risk of covid-19 ...” Participant 37

“…Creating awareness ...” Participant 38

“…At first, we thought it was a normal virus, just like another virus ...” Participant 46

“…Tells me how safe and secure from covid19 ...” Participant 47

“…keeps me alert, aware & informed ...” Participant 48

“…keeps me informed so that I can clarify doubts of non-medical persons ...” Participant 49

“…as far as the impact of information is concerned, I find it reliable and reassuring it is correct and updated regularly ...” Participant 51

“…precise news from tv and informative, credible sources on YouTube ...” Participant 53

“…this information is helping us to understand where India as a country, stands with respect to the world in terms of the fight against covid19. The Prime Minister's address helped us to understand the gravity of the situation and the importance of social distancing; also the information about India developing new testing kits has a positive impact on our minds. we as a country being united and staging a mature fight against covid 19 also helps to create positive vibes ...” Participant 54

“…the personal impact of information is predominantly positive as it helps us sensitize and prepare precautionary measures ...” Participant 55

**Neutral:**

The participants reported Neutral response as an outcome of the media information on COVID 19:

“…Doesn’t have too much of an impact, the impact is from being confined to my house ...” Participant 23

“…I'm just staying at home ...” Participant 29

“…Not really ...” Participant 42

“…In no ways ...” Participant 43

**Codes used for RQ1:**

*Label: Anxiety*

Definition: Anxiety is a feeling of fear, worry, or unease.

Description: Passage where participants have discussed fear, worry, or unease.

Inclusion criteria: People mention the feeling of fear, worry or unease.

Exclusion criteria: People mention of feeling relaxed.

Example: Uncertainty about the future, especially economic crisis.

*Label: Knowledge*

Definition: Knowledge consists of facts, information, and skills acquired through experience or education.

Description: Passages where Knowledge is discussed.

Inclusion criteria: People mention ignorance.

Exclusion criteria: People talk of ignorance.

Examples: Making aware of our surroundings.

*Label: Neutral*

Definition: Refers to an unbiased state or person

Description: Passage where an impartial response is mentioned.

Inclusion criteria: People mention neutral responses.

Exclusion criteria: People mention biased responses.

Examples: Doesn’t have too much of an impact.

**RQ 2: What is the impact of misinfodemics (spread of an epidemic/disease through misinformation) on social-psychology in India?**

Our analysis identified three themes; (1) Panic, (2) Confusion and (3) Distrust

**Panic:**

The participants reported that Panic, which includes feelings of fear or Anxiety as an impact of misinfodemics of the media information about COVID 19 on social psychology:

“…certain section may get infected and will also affect the country at large …” Participant 1

“…It may create Panic…” Participant 3

“…Yes myths undoubtedly increases fears and insecurities …” Participant 4

“…Increased risks, increased issues …” Participant 7

“…It will increase number of cases …” Participant 9

“…People become scared …” Participant 10

“…They are scared…” Participant 12

“…Panic, wrong self-medication, and even a false sense of security …” Participant 14

“…We are supposed to get the actual figure or a larger number, so that people here in India will be scared more so that people should be serious about lockdown …” Participant 17

“…Creates Panic and misunderstanding …” Participant 18

“…Panic and fear of death and frustration…” Participant 20

“…It creates havoc and chaos for no reason …” Participant 21

“…In the minds of fools, anything can create havoc …” Participant 24

“…It affects one’s health …” Participant 26

“…While we await concrete evidence for this claim from the scientific community, people are seeking hydroxychloroquine without realizing that it is a toxic drug and must be taken under supervision …” Participant 27

“…It will create more tension …” Participant 30

“…it’s immense as even the educated people of this country don’t have a scientific mindset…” Participant 32

“…People get on sided thoughts…” Participant 34

“…People are panicking …” Participant 39

“…It will get spread more due to these myths…” Participant 46

“…it does impact people who do not follow government instruction …” Participant 47

“…well as long as it doesn’t harm their health, they can do whatever they want, but it should not make them complacent …” Participant 48

“…people believe in all sorts of things or messages which don’t have any scientific base, they receive through WhatsApp, this is dangerous …” Participant 49

“…what’s more concerning is that when people believe these things and maybe we do that to some extent …. My friends and I often talk about how the masses would be reacting to the information, I often feel scared when I think about the fact that many won’t even come to know that they have Covid19 and by the time they come to know (if they come to know) it would be too late…” Participant 50

“…increase in stress anxiety and depression, the suicide of parents with their kids who were already struggling with financial and emotional distress, increase in hate towards a section of people …” Participant 51

“…Panic, misunderstanding of the precaution to be adapted …” Participant 55

“…it furthers the ideology which is anti-communist …” Participant 57

“…major issues of anxiety are misinformation …” Participant 58

“…they got anxious and agitated …” Participant 59

**Confusion:**

The participants reported that increased confusion as an impact of misinfodemics of the media information about COVID 19 on social psychology:

“…Misguide them ...” Participant 2

“…Some innocent people would simply follow the data and the suggestions and would feel they are safe. However, it impacts not just them but everyone around them.....ultimately having a cascading effect on society ...” Participant 8

“…Makes equipped with wrong info and raises expectations which when broken shatters them ...” Participant 13

“…People might act stupid or mislead others too ...” Participant 15

“…Risk of increasing confusion as well as disease ...” Participant 16

“…People wrong actions lead to results not as expected and hence spread ...” Participant 25

“…Normal population starts doing unuseful things ...” Participant 31

“…Some foolish people would experiment with their lives and also the lives of others ...” Participant 36

“…General masses consider this pandemic an easily curable subject & then lead a lenient life...” Participant 53

“…lack of control over themselves or others elders at home trying to fix and enforce them with many things while unable to do anything about the situation is also reported, feelings of uncertainty and not having a closure about many things is also lingering effect (many of my patients are college students) ...” Participant 56

**Distrust:**

The participants reported Distrust as a response as an impact of misinfodemics of the media information about COVID 19 on social psychology:

“…It motivates people to keep going but also causes unnecessary situations that can trigger the further spread of the pandemic ...” Participant 5

“…People will get careless about maintaining social distancing ...” Participant 6

“…Most of the peoples in the world lost their jobs, according to the current situation in world peoples didn’t get jobs easily, the poverty line will rise periodically which results in thefts and crimes will rise in each state ...” Participant 11

“…People have false hope, and the politicians are using this information to get the public to resume their jobs to get the economy up and about. While ignoring the huge human loss that may result from such misinformed decision ...” Participant 19

“…People start to consume these medicines, which can lead to further health problems ...” Participant 22

“…Some people will not take the virus as seriously ...” Participant 23

“…People without knowing the side effect of it have started purchasing it and taking and because of it has proved fatal ...” Participant 35

“…general masses may find it difficult to differentiate between true information and myths; hence media and the general public should be cautious while forwarding information ...” Participant 54

**Codes used for RQ2:**

*Label: Panic*

Definition: Panic is sudden uncontrollable fear or Anxiety, often causing wildly (lacks discipline) unthinking behaviour.

Description: Passage where participants have discussed about fear, undisciplined behaviour.

Inclusion criteria: People mention the feeling of fear, Anxiety.

Exclusion criteria: People mention of feeling relaxed.

Example: Creates Panic and misunderstanding.

*Label: Confusion*

Definition: Confusion means uncertainty about what is happening, intended or required.

Description: Passages where uncertainty is discussed.

Inclusion criteria: People mention misleading.

Exclusion criteria: People talk of certainty.

Examples: People might act stupid or mislead others too.

*Label: Distrust*

Definition: Refers to the feeling that someone or something cannot be relied upon.

Description: Passage where response related to doubting honesty or reliability is mentioned.

Inclusion criteria: People mention suspicion.

Exclusion criteria: People mention trust.

Examples: People start to consume these medicines, which can lead to further health problems.

**RQ 3: What is the impact of quarantine and isolation on the social-psychology of Indians battling COVID-19?**

Our analysis identified two themes; (1) Cognitive dissonance and (2) Adaptability.

**Cognitive dissonance:**

The participants reported experiencing cognitive dissonance, which includes experiencing two or more contradictory beliefs, ideas, or values as an impact of social distancing and self-quarantine when battling COVID-19:

“…For me, it does not impact much, but my children feel irritated because they want to play outside …” Participant 4

“…Personally, it is not impacting, but professionally I feel some monetary losses are going to happen …” Participant 6

“…It is not very healthy for normal function …” Participant 7

“…I have started getting restless now, and my mother seems to be getting impacted the most, leading to high sugar levels due to restlessness …” Participant 8

“…We are not able to do our jobs; there may be chances of getting fired from job because of the adverse impact on economic growth …” Participant 9

“…The relations are getting even better since we spend a lot of time together though it is creating problems as the requirements are not being fulfilled to a great extent …” Participant 13

“…All good except lack of grounding, I am desperate to feel the earth below my feet …” Participant 14

“…its good for few days, but for a long time, it disturbs all of us because I think now it's not a habit of leading their lives jointly …” Participant 16

“…Spending more time with family, but during working hours, it's stressful to handle multiple responsibilities, and with a lack of understanding of macro factors makes things worse than they are …” Participant 19

“…I am being inhuman and can't believe anyone …” Participant 20

“…All the work stations are not working, but the recurring expenditure is due …” Participant 21

“…I think Mental Health is being impacted. …” Participant 23

“…Because of social distancing, everything gets stop …” Participant 26

“…Difficult but managing, my business is affected, and major problem is income …” Participant 29

“…I know it’s needed, and I am following it diligently, and I may have to lose my job for this …” Participant 32

“…Emotional turmoil, depression does take over some time as we are not able to go out and meet our loved ones…” Participant 35

“…Feels restless and confused …” Participant 36

“…Increasing stress, Panic, meltdowns, and fights …” Participant 39

“…My work has come to a standstill leading to no income, and my son can’t go down to play …” Participant 42

“…it is creating a frustrating and depressing environment at home, and it is becoming difficult because everyone is facing their real self and has little or no escape as distraction from problems that were earlier available are now not available, on the other hand relationships are getting whole new meaning , different aspect of family members are visible …” Participant 51

“…acute pressure of living in the family scenario often personal spaces are being intruded getting impacted by family members issues with each other unable to have private space to have their sessions even …” Participant 56

“…The first week was ok, but from the second week, it’s a frustration to be in limited space and doing all work themselves …” Participant 57

**Adaptability:**

The participants reported that increased adaptability as an impact of social distancing and self-quarantine when battling COVID-19:

“…Lockdown is imposed based on government advisories, we don't go to the office or any other friend's place, but one has to get used to such lifestyle changes in such situations ...” Participant 5

“…It is not impacting self-isolation, and social distancing will protect my family...” Participant 11

“…It is a difficult time, but this time is being utilized for learning, alternative work, relaxation, meditation, etc. ...” Participant 15

“…We are spending a good time after so many years so that it may be boring but still different ...” Participant 17

“…Keeps us safe and secure and reduces the risk for others ...” Participant 18

“…Right now, it is the need of the hour, so no major impact ...” Participant 22

“…The disciplined family gets together, exploring new skills and knowledge, new experience, finishing pending to-do list ...” Participant 25

“…It has been a peaceful time for us as we live in a house (not an apartment), and we have the company of nature; we don't feel imprisoned at all, and we use this time creatively and constructively as I also work from home ...” Participant 27

“…The positive way we all are spending good time together ...” Participant 28

“…Initially it was tough, but now I have adapted ...” Participant 30

“…It is the need of the hour, so the impact is overweighed ...” Participant 34

“…about family, everything is still the same for me, but my bond between my close friend is improving due to the lockdown not much, however ...” Participant 44

“…when I returned home on 18^th^ March, the first time my mom did not hug me, she asked me to take a bath first...” Participant 48

“…it is helping my family members and me because we are together at home and communicating and talking is the key to stay positive ...” Participant 49

“…it’s making some bonds grow and giving us time to realize what is worth and what is not ...” Participant 52

“…nothing much as I am engrossed in my work, so I am already innovating new ways of doing business ...” Participant 53

“…it is helping the family to have good quality family time; we exercise together, we pray together, the children keep themselves busy by playing various games, helping with the household chores and reading books, they are also using their time to draw and paint ...” Participant 54

**Codes used for RQ3:**

*Label: Cognitive dissonance*

Definition: Cognitive dissonance occurs when a person holds two or more contradictory beliefs, ideas, or values.

Description: Passage where participants have discussed experiencing action that goes against their beliefs, values.

Inclusion criteria: People mention holding contradictory beliefs, ideas.

Exclusion criteria: People mention of feeling relaxed.

Example: I have started getting restless now, and my mother seems to be getting impacted the most, leading to high sugar levels due to restlessness.

*Label: Adaptability*

Definition: Adaptability is the quality of being able to adjust to new conditions

Description: Passages where people discuss ways of adaptability.

Inclusion criteria: People mention experiences of adjusting to the situation.

Exclusion criteria: People talk of non-adaptability.

Examples: Disciplined families get together, exploring new skills and knowledge, new experience, finishing pending to-do list.
