# Supplementary material for: Panic During COVID-19 Pandemic! A Qualitative Investigation Into the Psychosocial Experiences of a Sample of Indian People
Source: Front Psychol. 2020 Oct 15;11:575491. doi: 10.3389/fpsyg.2020.575491 (PMC7594525; doi:10.3389/fpsyg.2020.575491)
Supplement: Supplementary file 2 [file Table_2.DOCX]

**Annexure 2**

**COREQ (COnsolidated criteria for REporting Qualitative research) Checklist**

A checklist of items that should be included in reports of qualitative research. If you have not included this information, either revise your manuscript accordingly before submitting or note N/A.

| **Topic** | **Item No.** | **Guide Questions/Description** | **Response** |
| --- | --- | --- | --- |
| **Domain 1: Research team and reflexivity** | | |  |
| *Personal characteristics* |  |  |  |
| Interviewer/facilitator | 1 | Which author/s conducted the interview or focus group? | Author 6 |
| Credentials | 2 | What were the researcher’s credentials? | Master of Arts (Clinical Psychology), Master of Philosophy (Clinical Psychology) |
| Occupation | 3 | What was their occupation at the time of the study? | Clinical Psychologist |
| Gender | 4 | Was the researcher male or female? | Female |
| Experience and training | 5 | What experience or training did the researcher have? | Trained clinical psychologist, Experience of twelve years as clinical psychologist |
| *Relationship with participants* | | |  |
| Relationship established | 6 | Was a relationship established prior to study commencement? | Yes |
| Participant knowledge of the interviewer | 7 | What did the participants know about the researcher? | Her occupation of clinical psychologist, and reasons for carrying out this research |
| Interviewer characteristics | 8 | What characteristics were reported about the inter viewer/facilitator? | Occupation, reasons and interests in this research |
| **Domain 2: Study design** |  |  |  |
| *Theoretical framework* |  |  |  |
| Methodological orientation and Theory | 9 | What methodological orientation was stated to underpin the study? | Grounded theory |
| *Participant selection* |  |  |  |
| Sampling | 10 | How were participants selected? | Purposive |
| Method of approach | 11 | How were participants approached? | Telephone as well as email (face-to-face was not possible due to lockdown across the country) |
| Sample size | 12 | How many participants were in the study? | 59 (excluding drop-outs) |
| Non-participation | 13 | How many people refused to participate or dropped out? Reasons? | 21. Some of them refused to participate due to panic, while in some cases, the responses were not enough to be included in this qualitative research |
| *Setting* |  |  |  |
| Setting of data collection | 14 | Where was the data collected? | Home (it was work-from-home for the researchers during this lockdown period) |
| Presence of nonparticipants | 15 | Was anyone else present besides the participants and researchers? | Since the data were collected electronically, this question is not applicable |
| Description of sample | 16 | What are the important characteristics of the sample? | Twelve psychologists, nine service sector professionals, nine software engineers, four businesspersons, three financial consultants, six quarantined people (including two Covid-19 patients), three media persons, two artists, two educationists, two social workers, one spiritual healer, one doctor, one graphic designer, one housekeeping supervisor, one defence person, one banker, one insurance advisor |
| *Data collection* |  |  |  |
| Interview guide | 17 | Were questions, prompts, guides provided by the authors? Was it pilot tested? | Questions were provided, no prompts or guides were provided. Questions were pilot tested on 6 participants other than the subjects included in the study |
| Repeat interviews | 18 | Were repeat interviews carried out? If yes, how many? | Yes. 3 interviews had to be repeated. |
| Audio/visual recording | 19 | Did the research use audio or visual recording to collect the data? | No |
| Field notes | 20 | Were field notes made during and/or after the inter view or focus group? | Yes, field notes were made |
| Duration | 21 | What was the duration of the inter views or focus group? | 30-45 minutes |
| Data saturation | 22 | Was data saturation discussed? | Yes. Data saturation has been discussed |
| Transcripts returned | 23 | Were transcripts returned to participants for comment and/or correction? | Yes, for the three repeat interviews. |
| **Domain 3: analysis and findings** | | |  |
| *Data analysis* |  |  |  |
| Number of data coders | 24 | How many data coders coded the data? | Author 2 and Author 3 |
| Description of the coding tree | 25 | Did authors provide a description of the coding tree? | Yes (provided in the supplementary file ‘Annexure 1’) |
| Derivation of themes | 26 | Were themes identified in advance or derived from the data? | Derived from the data |
| Software | 27 | What software, if applicable, was used to manage the data? | NVIVO12 |
| Participant checking | 28 | Did participants provide feedback on the findings? | No |
| *Reporting* |  |  |  |
| Quotations presented | 29 | Were participant quotations presented to illustrate the themes/findings?  Was each quotation identified? | Yes (provided in the supplementary file ‘Annexure 1’) |
| Data and findings consistent | 30 | Was there consistency between the data presented and the findings? | Yes |
| Clarity of major themes | 31 | Were major themes clearly presented in the findings? | Yes |
| Clarity of minor themes | 32 | Is there a description of diverse cases or discussion of minor themes? | Yes |

Developed from: Tong A, Sainsbury P, Craig J. Consolidated criteria for reporting qualitative research (COREQ): a 32-item checklist for interviews and focus groups. *International Journal for Quality in Health Care*. 2007. Volume 19, Number 6: pp. 349 – 357

**Once you have completed this checklist, please save a copy and upload it as part of your submission. DO NOT** **include this checklist as part of the main manuscript document. It must be uploaded as a separate file.**
